# Supplementary material for: LC/MS-MS Analysis of Phenolic Compounds in Hyoscyamus albus L. Extract: In Vitro Antidiabetic Activity, In Silico Molecular Docking, and In Vivo Investigation against STZ-Induced Diabetic Mice
Source: Pharmaceuticals (Basel). 2023 Jul 18;16(7):1015. doi: 10.3390/ph16071015 (PMC10384095; doi:10.3390/ph16071015)
Supplement: Supplementary file 1 [file pharmaceuticals-16-01015-s001.zip › pharmaceuticals-2409656-supplementary.pdf]

Supplementary Materials

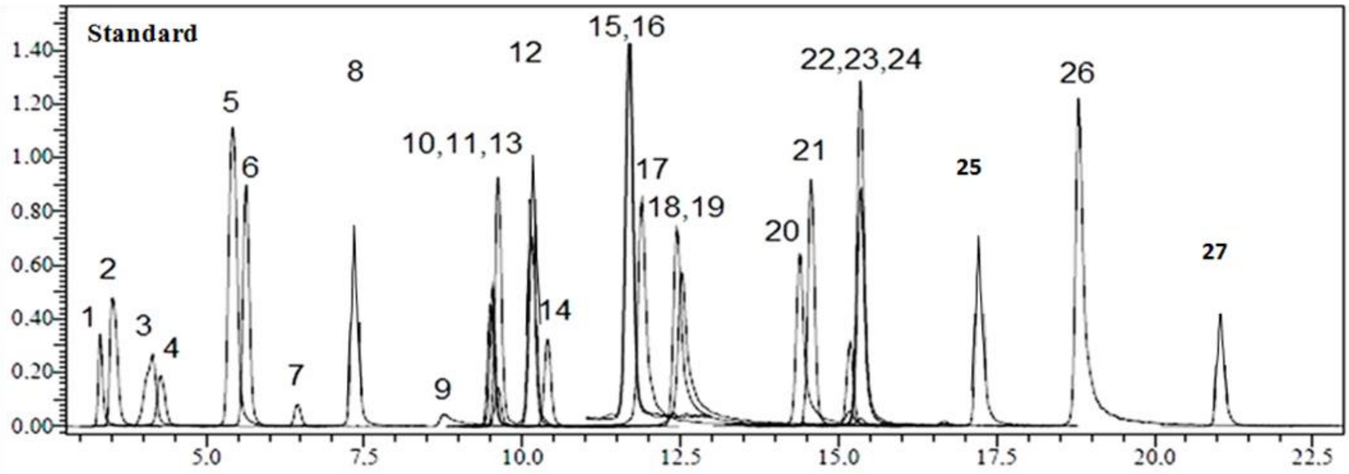

Figure S1. LC-MS/MS chromatograms of 250 ppb standard mix. Notes: Chromatographicconditionsweregiveninexperimentalsection.Standardcompounds:1:quinicacid,2:malicacid, 3:tr-aconiticacid,4:gallicacid,5:chlorogenicacid,6:protocatechuicacid,7:tannic acid,8:tr-caffeicacid,9:vanillin,10:*p*-coumaricacid,11:rosmarinicacid,12:rutin,13: hesperidin,14:hyperoside,15:4-OHbenzoicacid,16:salicylicacid,17:myricetin,18:fisetin, 19:coumarin,20:quercetin,21:naringenin,22:hesperetin,23:luteolin,24:kaempferol,25: apigenin,26:rhamnetin,27:chrysin.

Table S1. AnalyticalparametersofLC-MS/MSmethod

	Analyte	RT	R^2	RSD%	LinearityRange(mg/L)	LOD/LOQ (μg/L)	Recovery(%)	
1	**Quinicacid**	3.32	0.9927	0.0388	250–10,000	22.3/74.5	103.3	4.8
2	**Malic acid**	3.54	0.9975	0.1214	250–10,000	19.2/64.1	101.4	5.3
3	**tr-Aconiticacid**	4.13	0.9933	0.3908	250–10,000	15.6/51.9	102.8	4.9
4	**Gallic acid**	4.29	0.9901	0.4734	25–1000	4.8/15.9	102.3	5.1
5	**Chlorogenic acid**	5.43	0.9932	0.1882	250–10,000	7.3/24.3	99.7	4.9
6	**Protocatechuicacid**	5.63	0.9991	0.5958	100–4000	25.8/85.9	100.2	5.1
7	**Tannic acid**	6.46	0.9955	0.9075	100–4000	10.2/34.2	97.8	5.1
8	**tr-Caffeic acid**	7.37	0.9942	1.0080	25–1000	4.4/14.7	98.6	5.2
9	**Vanillin**	8.77	0.9995	0.4094	250–10,000	10.1/33.7	99.2	4.9
10	***p*-Coumaric acid**	9.53	0.9909	1.1358	100–4000	15.2/50.8	98.4	5.1
11	**Rosmarinic acid**	9.57	0.9992	0.5220	250–10,000	10.4/34.8	101.7	4.9
12	**Rutin**	10.18	0.9971	0.8146	250–10,000	17.0/56.6	102.2	5.0
13	**Hesperidin**	9.69	0.9973	0.1363	250–10,000	21.6/71.9	100.2	4.9
14	**Hyperoside**	10.43	0.9949	0.2135	100–4000	12.4/41.4	98.5	4.9
15	**4-OHBenzoicacid**	11.72	0.9925	1.4013	25–1000	3.0/10.0	106.2	5.2
16	**Salicylicacid**	11.72	0.9904	0.6619	25–1000	4/13.3	106.2	5.0
17	**Myricetin**	11.94	0.9991	2.8247	100–4000	9.9/32.9	106.0	5.9
18	**Fisetin**	12.61	0.9988	2.4262	100–4000	10.7/35.6	96.9	5.5
19	**Coumarin**	12.52	0.9924	0.4203	100–4000	9.1/30.4	104.4	4.9
20	**Quercetin**	14.48	0.9995	4.3149	25–1000	2.0/6.8	98.9	7.1
21	**Naringenin**	14.66	0.9956	2.0200	25–1000	2.6/8.8	97.0	5.5

**22**	**Hesperetin**	15.29	0.9961	1.0164	25–1000	3.3/11.0	102.4	5.3
**23**	**Luteolin**	15.43	0.9992	3.9487	25–1000	5.8/19.4	105.4	6.9
**24**	**Kaempferol**	15.43	0.9917	0.5885	25–1000	2.0/6.6	99.1	5.2
**25**	**Apigenin**	17.31	0.9954	0.6782	25–1000	0.1/0.3	98.9	5.3
**26**	**Rhamnetin**	18.94	0.9994	2.5678	25–1000	0.2/0.7	100.8	6.1
**27**	**Chrysin**	21.18	0.9965	1.5530	25–1000	0.05/0.17	102.2	5.3

Parent ion (*m/z):* Molecular ions of the standard compounds (mass to charge ratio); MS^2 (CE): MRM fragments for the related molecular ions (CE refers to related collision energies of thefragment ions); RT: Retention time; R^2: coefficient of determination; RSD: relative standard deviation; LOD/LOQ (µg/L): Limit of detection/Limit of quantification.

Table S2. Drug-likeness characteristics of the acarbose and the top active ligands of *H. albus*

	**MW g/mol**	**logP**	**Log S**	**HBA**	**HBD**	**TPSA (Å^2)**	**AMR**	**nRB**	**Lipinski**	**Ghose**	**Veber**	**Egan**
Acarbose	645.60	-6.22	2.13	19	14	321.17	136.69	9	No	No	No	No
Luteolin	286.24	1.73	-3.71	6	4	111.13	76.01	1	Yes	Yes	Yes	Yes
Fisetin	286.24	1.55	-3.35	6	4	111.13	76.01	1	Yes	Yes	Yes	Yes
Rutin	610.52	-1.29	-3.30	16	10	269.43	141.38	6	No	No	No	No
Qercetin	302.24	1.23	-3.16	7	5	131.36	78.03	1	Yes	Yes	Yes	Yes

AMR: Atom Molar Refractivity; nRB; Num. rotatable bonds; HBD: Num. H-bond donors; HBA: Num. H-bond acceptors.

Table S3. ADMET properties of the acarbose and the top active ligands of *H. albus*

	**Criteria**	**Acarbose**	**Luteolin**	**Fisetin**	**Rutin**	**Quercetin**
Absorption	BBB penetration	N	N	N	N	N
Distribution	Caco2	L	H	H	L	L
	HIA	L	H	H	L	H
Metabolism	CYP1A2 inhibitor	N	Y	Y	N	Y
	CYP2C19 inhibitor	N	N	N	N	N
	CYP2C9 inhibitor	N	N	N	N	N
	CYP2D6 inhibitor	N	Y	Y	N	Y
	CYP3A4 inhibitor	N	Y	Y	N	Y
Excretion	Cl	L	M	M	L	M
Toxicity	hERGBlockers	N	N	N	N	N
	AMES Toxicity	N	Y	Y	N	Y
	Skin Sensitization	N	Y	Y	N	Y
	Carcinogenicity	N	N	N	N	Y
	Cytotoxicity	N	N	N	N	N
	Immunotoxicity	N	N	N	Y	N
	H-HT	Y	N	N	N	N
	Eye Corrosion	N	N	N	N	N
	Respiratory toxicity	N	N	N	N	N
	NR-AR	N	N	N	N	N
	NR-ER	Y	Y	Y	N	Y
	SR-p53	N	N	N	N	N

BBB, Blood-Brain Barrier. HIA, Human Intestinal Absorption. Caco2, Permeability assay. hERG, human Ether-a-go-go-Related Gene potassium channel. H-HT, Human Hepatotoxicity. NR-AR, Androgen receptor disruptor. NR-ER, Estrogen receptor disruptor. SR-p53, tumor suppressor protein p53 activator. Cl, clearance of the molecule. N: inactive. Y: active. L: Low. M: Moderate. H: High.
